# Serum anti-PCK1 antibody levels are a prognostic factor for patients with diabetes mellitus

**DOI:** 10.1186/s12902-023-01491-3

**Published:** 2023-10-30

**Authors:** Toshiki Namiki, Minoru Takemoto, Aiko Hayashi, Hiroki Yamagata, Takahiro Ishikawa, Koutaro Yokote, Shu-Yang Li, Masaaki Kubota, Bo-Shi Zhang, Yoichi Yoshida, Tomoo Matsutani, Seiichiro Mine, Toshio Machida, Yoshio Kobayashi, Jiro Terada, Akira Naito, Koichiro Tatsumi, Hirotaka Takizawa, Rika Nakamura, Hideyuki Kuroda, Yasuo Iwadate, Takaki Hiwasa

**Affiliations:** 1 Department of Diabetes, Metabolism and Endocrinology, School of Medicine, International University of Health and Welfare, Narita Hospital, 852 Hatakeda, Narita City, Chiba, 286-8520 Japan; 2 Department of Endocrinology, Hematology and Gerontology, Graduate School of Medicine, Chiba University, Chiba, 260-8670 Japan; 3 Department of Neurological Surgery, Graduate School of Medicine, Chiba University, Chiba, 260-8670 Japan; 4 Department of Neurological Surgery, Chiba Prefectural Sawara Hospital, Chiba, 287-0003 Japan; 5 Department of Neurological Surgery, Chiba Cerebral and Cardiovascular Center, Chiba, 290-0512 Japan; 6 Department of Neurosurgery, Eastern Chiba Medical Center, Chiba, 283-8686 Japan; 7 Department of Cardiovascular Medicine, Graduate School of Medicine, Chiba University, Chiba, 260-8670 Japan; 8 Department of Respirology, Graduate School of Medicine, Chiba University, Chiba, 260-8670 Japan; 9 Port Square Kashiwado Clinic, Kashiwado Memorial Foundation, Chiba, 260-0025 Japan; 10 Medical Project Division, Research Development Center, Fujikura Kasei Co, Saitama, 340-0203 Japan

**Keywords:** Phosphoenolpyruvate carboxykinase 1, Diabetes mellitus, Atherosclerosis, Cardiovascular disease, Antibody biomarker

## Abstract

**Background:**

Autoantibodies develop in autoimmune diseases, cancer, diabetes mellitus (DM), and atherosclerosis-related diseases. However, autoantibody biomarkers have not been successfully examined for diagnosis and therapy.

**Methods:**

Serological identification of antigens through recombinant cDNA expression cloning (SEREX) was used for primary screening of antigens. The cDNA product was expressed in bacteria and purified. Amplified luminescent proximity homogeneous assay-linked immunosorbent assay (AlphaLISA) was used to evaluate antibody levels in serum samples.

**Results:**

Phosphoenolpyruvate carboxykinase 1 (PCK1) was recognized as an antigen by serum IgG antibodies in the sera of patients with atherosclerosis. AlphaLISA showed significantly higher serum antibody levels against recombinant PCK1 protein in patients with DM and cardiovascular disease than in healthy donors, but not in those with acute ischemic stroke, transient ischemic attack, or obstructive sleep apnea syndrome. The area under the receiver operating characteristic curve for anti-PCK1 antibodies was 0.7024 for DM. The serum anti-PCK1 antibody levels were associated with age, platelet count, and blood pressure. Anti-PCK1-antibody-positive patients showed significantly lower overall survival than the negative patients.

**Conclusions:**

Serum anti-PCK1 antibody levels were found to be associated with DM. The anti-PCK1 antibody marker is useful for predicting the overall survival of patients with DM.

**Supplementary Information:**

The online version contains supplementary material available at 10.1186/s12902-023-01491-3.

## Background

The number of patients with diabetes mellitus (DM) has considerably increased worldwide, which is now referred to as the diabetes pandemic [1]. DM is a disease in which blood glucose levels increase owing to decreased insulin secretion or increased resistance. Prolonged high blood glucose levels increase the risk of diabetic complications such as acute ischemic stroke (AIS) and cardiovascular disease (CVD), which are mainly caused by the development of atherosclerosis [2]. Early treatment of DM is important, not only to prevent diabetic complications, but also to prolong the life span of patients with DM [3].

We screened autoantibodies in the sera of patients with atherosclerosis using serological analysis of recombinant cDNA expression libraries (SEREX) and protein array methods. The following autoantibodies have been identified as biomarkers for atherosclerotic diseases including AIS [4, 5] and CVD [6–8]: matrix metalloproteinase 1 [9], adaptor-related protein complex 3 subunit delta 1 [10], forkhead box J2 [11], and bone morphogenetic protein 1 [12]. It is not surprising that these autoantibodies are also associated with DM, as there is a strong association between atherosclerosis and DM [13].

In this study, we identified phosphoenolpyruvate carboxykinase 1 (PCK1, also known as PEPCK) by SEREX screening using sera from patients with atherosclerosis and found that its autoantibody levels were specifically elevated in patients with DM. The anti-PCK1 antibody marker is useful for predicting the overall survival of patients with DM. Furthermore, the possible relevance of PCK1 activity in DM development is discussed.

## Methods

### Patient and control sera

The protocols of this study complied with the 1975 Declaration of Helsinki and were approved by the Local Ethical Review Board of Chiba University, Graduate School of Medicine in Chiba, Japan (No. 2017–251, 2018–320, 2020–1129), as well as by the Review Boards of the participating hospitals. Sera were collected from patients who provided written, informed consent. Each serum sample was centrifuged at 3000 × *g* for 10 min, and the supernatant was stored at − 80 °C until use, avoiding repeated freezing/thawing of samples.

Serum samples from 275 patients with DM, 85 patients with CVD, and 86 patients with obstructive sleep apnea syndrome (OSAS) were obtained from the Chiba University Hospital. Samples collected from 228 patients with AIS and 44 with transient ischemic attack (TIA) were obtained from Chiba Prefectural Sawara Hospital. Serum samples from patients with AIS, TIA, and CVD were obtained within 2 weeks of disease onset. Serum samples from healthy donors (HDs) were obtained from the Port Square Kashiwado Clinic and Chiba Prefectural Sawara Hospital. These HD participants were selected from those that exhibited no abnormalities on cranial magnetic resonance imaging for comparison with TIA and AIS. We compared patients with DM to 81 HDs, patients with OSAS and CVD to 76 HDs, and patients with TIA or AIS to 138 HDs. HDs selected for comparison had blood samples collected at approximately the same time as the samples from each respective disease group.

The patients were randomly selected, and the definition of each disease is as follows. (1) DM was diagnosed according to the diagnostic criteria of the Japanese Diabetes Society [14]. Moreover, patients who had already been prescribed oral hypoglycemia agents and/or insulin injections were considered patients with DM. (2) Patients with CVD were those who visited the emergency medical department due to acute myocardial infarction or unstable angina pectoris. (3) OSAS was diagnosed by polysomnography (PSG) [15]. (4) For AIS, TIA, the stroke subtype of each patient was also determined according to the criteria of the Trial of Org 10,172 in the Acute Stroke Treatment classification system [16]. In this analysis, large-artery atherosclerosis or small-artery occlusion (lacune) were considered AIS or cerebral infarction [11].

The patients with diabetes were followed up for 100 months. Each patient’s status was checked on the electric medical record on all hospital visits.

### SEREX screening

To select the antigens recognized by serum IgG antibodies, SEREX screening was performed using sera from patients with atherosclerosis and a human aortic endothelial cell cDNA phage library (Uni-ZAP XR Premade Library, Stratagene, La Jolla, CA), as described previously [17]. The SEREX process is a well-established technique for pinpointing antigenic proteins. SEREX merges molecular cloning via phage expression libraries with serological typing, making it a highly efficient and user-friendly approach for identifying antigenic markers across the human genome. This method has successfully uncovered over 1000 novel tumor antigens and is widely regarded as a powerful tool for identifying potential targets in various forms of malignant tumors. We searched for antibody markers supposedly associated with atherosclerosis using SEREX. We identified approximately 100 different antibodies that might be related to atherosclerotic vascular disease, some of which had been already reported. PCK1-Ab was identified through this screening. As diabetes seems to be closely related to the development and progression of atherosclerotic vascular disease, we analyzed the relationship between PCK1-Ab and DM.

### Preparation of recombinant PCK1 protein

We cloned the human PCK1 cDNA sequence (accession number: NP_001284695.1) into the *Eco*RI/*Xho*I site of pGEX-4 T-1 (Cytiva, Pittsburgh, PA, USA). We induced the expression of the cDNA product by treating *Escherichia coli* BL-21 cells harboring the pGEX-4 T-1-PCK1 with 0.1 mM of isopropyl-β-D-thiogalactoside (Wako Pure Chemicals, Osaka, Japan) at 37 °C for 3 h. Cells were lysed by sonication in the BugBuster Master Mix (Merck Millipore, Darmstadt, Germany). Glutathione S-transferase (GST)-fused PCK1 protein was purified using Glutathione-Sepharose 4 Fast Flow medium (Cytiva) and then concentrated to 1.56 mg/mL in phosphate-buffered saline as described previously [9, 10].

The GST-fused full-length protein was expressed in bacteria and purified by affinity chromatography, as previously described [9].

### Amplified luminescence proximity homogeneous assay-linked immunosorbent assay (AlphaLISA)

AlphaLISA was performed in 384-well microtiter plates (white opaque OptiPlate™, Perkin Elmer, Waltham, MA, USA), containing either 2.5 µL of 1:100-diluted serum with 2.5 µL of GST or GST-PCK1 proteins (10 µg/mL) in AlphaLISA buffer [25 mM *N*-(2-hydroxyethyl) piperazine-*N*ʹ-2-ethane sulfonic acid, pH 7.4, 0.1% casein, 0.5% Triton X-100, 1 mg/mL dextran-500, and 0.05% ProClin-300]. We incubated the reaction mixture at room temperature for 6–8 h, and then added anti-human IgG-conjugated acceptor beads (2.5 µL at 40 µg/mL) and glutathione-conjugated donor beads (2.5 µL at 40 µg/mL). The mixture was then incubated at room temperature in the dark for 7–21 days. We measured the chemical emissions using an EnSpire Alpha microplate reader (PerkinElmer), as described previously [8, 9, 17, 18]. We calculated the specific reactions by subtracting the emission photon counts of the GST control from the counts of the GST-fused PCK1 protein.

AlphaLISA is a novel, recently developed method. After examining suitable AlphaLISA conditions in this study, we concluded that incubation for 7–21 days is the best option to obtain specific antigen-Ab reaction as well as to reduce background noise.

### Statistical analysis

We employed the Mann–Whitney U test to determine the significant differences between the two groups, and the Kruskal–Wallis test (Mann–Whitney U test with Bonferroni correction applied) to evaluate the differences among three groups. Correlations were calculated using Spearman’s correlation. All statistical analyses were performed using GraphPad Prism 5 (GraphPad Software Inc., La Jolla, CA, USA). We assessed the predictive values of the putative disease markers via receiver operating characteristic (ROC) curve analysis and set the cutoff values of the Youden index, which maximizes the sum of sensitivity and specificity. We evaluated patient survival using the Kaplan–Meier method and compared them using the log-rank test. All tests were 2-tailed, and *P* values < 0.05 were considered to indicate statistically significant differences.

## Results

### Recognition of PCK1 by serum antibodies in patients with atherosclerosis

We performed SEREX screening and identified PCK1 (Accession Number: NM_002591.4) as the antigen recognized by antibodies in the sera of patients with atherosclerosis. Subsequently, the GST-fused full-length PCK1 protein and control GST protein were expressed in bacteria and purified by affinity chromatography. The results of sodium dodecyl-sulfate–polyacrylamide gel electrophoresis showed that the purity of both GST-PCK1 and GST was higher than 95% (Supplementary Figure S1).

### Elevated levels of serum anti-PCK1 antibodies (s-PCK1-Abs) in patients with DM

**Fig. 1:**
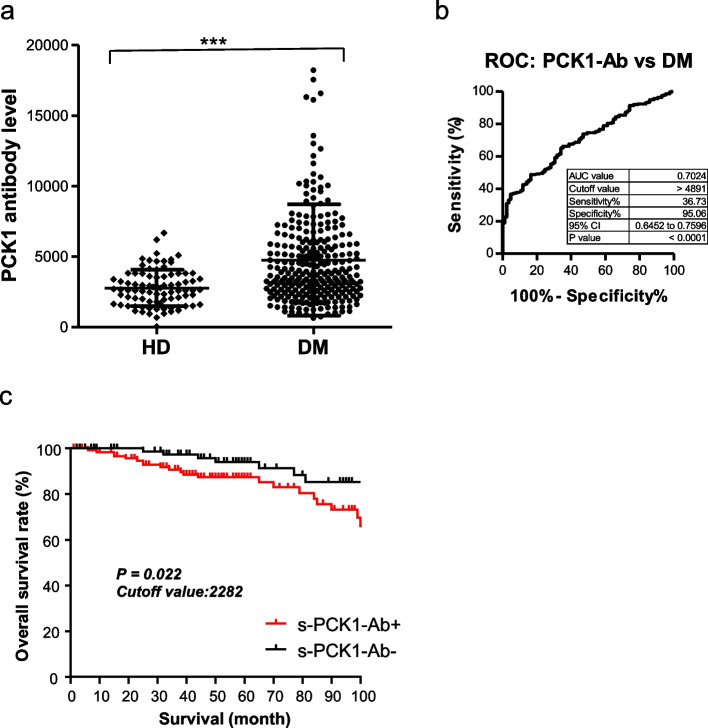
Comparison of s-PCK1-Ab levels between healthy donors (HDs) and patients with diabetes mellitus (DM). **a** The serum anti-PCK1 antibody (s-PCK1-Ab) levels of HDs and patients with DM were examined by AlphaLISA using GST-PCK1_2-302_ protein as the antigen, followed by subtraction of the levels against control GST. A scatter dot plot of the s-PCK1-Ab levels is shown. The bars represent the average and the average ± SD. *P* values were calculated using the Kruskal–Wallis test. *** *P* < 0.001 vs. HD specimens. The total (male/female) numbers, average ages ± standard deviations (SDs), average antibody levels ± SDs, cutoff values, positive numbers, positive rates (%), and *P* values versus HDs are summarized and shown in Table 1. **b** Receiver operating characteristic (ROC) curve analysis. The ability of s-PCK1-Abs to detect DM was evaluated using ROC curve analysis. Numbers in the figures indicate the areas under the ROC curve (AUC), cutoff values for antibody levels, sensitivity, specificity, and 95% confidence interval (95% CI). **c** Comparison of the overall survival in patients with DM between the positive and negative groups of s-PCK1-Abs. Kaplan–Meier with the log-rank test is shown

Next, we examined s-PCK1-Ab levels in patients with DM using GST-PCK1 as an antigen. Serum samples from 81 HDs and 275 patients with DM were obtained from Port Square Kashiwado Clinic and Chiba University Hospital, respectively. The s-PCK1-Ab levels were significantly higher in patients with DM than in HDs (Fig. 1a). Using the cutoff values of the average plus two standard deviations (SDs) of the HD values, the positive rates of s-PCK1-Abs in HDs and patients with DM were 2.5% and 29.5%, respectively (Table 1). We performed ROC analysis to evaluate the ability of the s-PCK1-Ab marker to indicate the presence of DM. The area under the ROC curve (AUC) for s-PCK1-Abs was 0.7024, yielding sensitivity and specificity of 36.73% and 95.06%, respectively (Fig. 1b).

**Table 1:** Basic characteristics and positive rates of s-PCK1-Abs in HDs and patients with DM

Sample information		HDs	DM
	Total sample number	81	275
	Male/Female	46/35	158/117
	Type 1 DM / Type 2 DM	-	26/216
	Age (Average ± SD)	45.2 ± 11.0	63.1 ± 12.0
Subject group	Type of value	s-PCK1-Ab	
HDs	Average	2,831	
	SD	1,298	
	Cutoff value	5,427	
	Positive number	2	
	Positive rate (%)	2.5%	
DM	Average	4,766	
	SD	3,954	
	Positive number	81	
	Positive rate (%)	29.5%*	
	*P* (DM vs HDs)	< 0.001*	

*s-PCK-Ab* Serum phosphoenolpyruvate carboxykinase 1 antibody, *HD* Healthy donor, *DM* Diabetes mellitus, *SD* Standard deviation

^*^Significant correlations (*P* < 0.05) and positive rates (> 10%) are indicated in bold

The overall survival during the follow-up period of 100 months was compared between the s-PCK1-Ab-positive and -negative DM groups with a cutoff value of the Youden index. Thirty-three deaths were confirmed (99.6% of the cases were followed up). Two patients died owing to myocardial infarction, one owing to cerebral infarctions, and 12 owing to cancer. The causes of death were unknown for 18 cases.

The s-PCK1-Ab-positive group showed a more unfavorable prognosis than the negative group (*P* = 0.022) (Fig. 1c). It should be noted that the difference was more evident in the late stage (after 70 months) than in the early stage (before 60 months).

### Levels of s-PCK1-Abs in the patients with atherosclerosis-related diseases

**Fig. 2:**
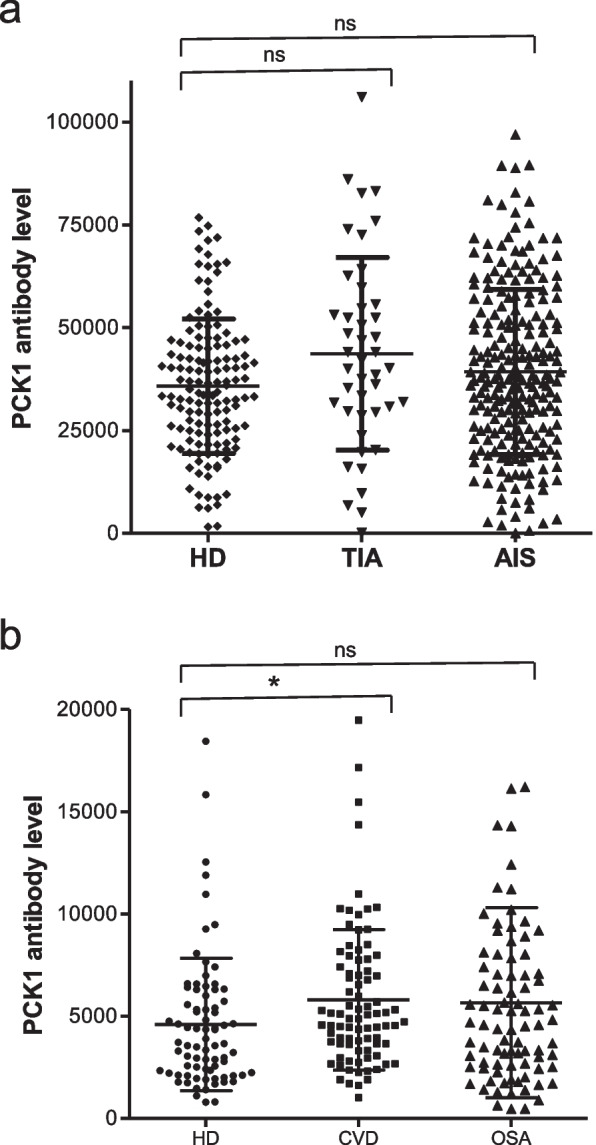
Comparison of serum s-PCK1-Ab levels between healthy donors (HDs) and patients. The s-PCK1-Ab levels of HDs and patients with transient ischemic attack (TIA) or acute ischemic stroke (AIS) (**a**) and cardiovascular disease (CVD) or obstructive sleep apnea syndrome (OSAS), (**b**) were examined by AlphaLISA using glutathione S-transferase (GST)-PCK1_2-302_ protein as the antigen, followed by subtraction of the levels against control GST. A scatter dot plot of the s-PCK1-Ab levels is shown as described in the legends of Fig. 1. The bars represent the average and average ± SD. *P* values were calculated using the Kruskal–Wallis test. *, *P* < 0.05 vs. HD specimens. ns, not significant. The total (male/female) numbers, average ages ± standard deviations (SDs), average antibody levels ± SDs, cutoff values, positive numbers, positive rates (%), and *P* values versus HDs are summarized and shown in Tables 2 and 3

PCK1 was screened by SEREX using sera from patients with atherosclerosis, so the antibody levels in other atherosclerosis-related diseases were also examined. We first examined the s-PCK1-Ab levels in patients with AIS or TIA. Sera from HDs and patients with AIS and TIA were obtained from Chiba Prefectural Sawara Hospital. AlphaLISA results revealed that s-PCK1-Ab levels were not significantly different between patients with AIS or TIA and HDs (Fig. 2a). At a cutoff value equivalent to the average plus two SDs of the HD values, the s-PCK1-Ab-positive rates for the HDs, patients with AIS, and those with TIA were 2.9%, 15.9%, and 8.8%, respectively (Table 2). Thus, a slight increase in positivity was observed in AIS and TIA.

**Table 2:** s-PCK1-Ab levels in the patients with TIA or AIS

Sample information		HD	TIA	AIS
	Total sample number	138	44	228
	Male/Female	86/52	24/20	129/99
	Age (Average ± SD)	51.8 ± 12.7	68.5 ± 12.1	77.0 ± 11.1
Subject group	Type of value	s-PCK1-Ab		
HD	Average	35,813		
	SD	16,346		
	Cutoff value	68,504		
	Positive number	4		
	Positive rate (%)	2.9%		
TIA	Average	43,677		
	SD	23,398		
	Positive number	7		
	Positive rate (%)	15.9%*		
	*P* (TIA vs HDs)	ns		
AIS	Average	39,250		
	SD	20,058		
	Positive number	20		
	Positive rate (%)	8.8%		
	*P* (AIS vs HDs)	ns		

*s-PCK-Ab* Serum phosphoenolpyruvate carboxykinase 1 antibody, *HD* Healthy donor, *DM* Diabetes mellitus, *TIA* Transient ischemic attack, *AIS* Acute ischemic stroke, *SD* Standard deviation, *ns* not significant

*A positive rate (> 10%) is indicated in bold

Atherosclerosis is a major risk factor for CVD, and OSAS is frequently accompanied by hypertension leading to atherosclerosis [6, 7, 16]. We examined antibody levels in serum samples from patients with CVD or OSAS obtained from Chiba University Hospital. The s-PCK1-Ab levels were slightly but significantly higher in the patients with CVD, but not in those with OSAS, as compared with those in HDs (Fig. 2b). At a cutoff value of the average plus two SDs of the HD samples, the positivity rates of HDs and patients with CVD and OSAS were 5.3%, 4.7%, and 9.3%, respectively (Table 3). Thus, c-PCK1-Abs may not be closely associated with CVD or OSAS.

**Table 3:** s-PCK1-Ab levels with HDs and patients with CVD and OSAS

Sample information		HDs	CVD	OSAS
	Total sample number	76	85	86
	Male/Female	43/33	70/15	59/27
	Age (Average ± SD)	45.1 ± 11.5	66.4 ± 11.6	57.8 ± 12.5
Subject group	Type of value	s-PCK1-Ab		
HD	Average	4,658		
	SD	3,260		
	Cutoff value	11,179		
	Positive number	4		
	Positive rate (%)	5.3%		
CVD	Average	5,805		
	SD	3,440		
	Positive number	4		
	Positive rate (%)	4.7%		
	*P* (CVD vs HDs)	< 0.05*		
OSAS	Average	5,656		
	SD	4,647		
	Positive number	8		
	Positive rate (%)	9.3%		
	*P* (OSAS vs HDs)	ns		

*s-PCK-Ab* Serum phosphoenolpyruvate carboxykinase 1 antibody, *HD* Healthy donor, *CVD* Cardiovascular disease, *OSAS* Obstructive sleep apnea syndrome, *SD* Standard deviation, *ns* Not significant

^*^A significant correlation (*P* < 0.05) is indicated in bold

### Correlation analysis

We performed a relation analysis between s-PCK1-Ab levels and participant data using 275 specimens from the DM cohort at Chiba University Hospital. In this analysis, we employed the Mann–Whitney U test to compare s-PCK1-Ab levels between male and female participants, type-1 and type-2 DM, with or without obesity (body mass index [BMI] ≥ 25), hypertension, CVD, dyslipidemia, and smoking or alcohol intake habits. We observed that s-PCK1-Ab levels were significantly higher in patients with hypertension than in those without (Table 4).

**Table 4:** Relation analysis of antibody levels against PCK1 protein with data of subjects in DM cohort

Sex		Male	Female
Sample number		156	119
s-PCK1-Ab level	Average	4,823	4,692
	SD	4,588	2,940
*P* value (vs Male)			0.5812
DM type		Type-1 DM	Type-2 DM
Sample number		28	215
s-PCK1-Ab level	Average	3,866	5,058
	SD	2,462	4,271
*P* value (vs BMI < 25)			0.0642
Obesity		BMI < 25	BMI ≥ 25
Sample number		156	114
s-PCK1-Ab level	Average	5,066	4,379
	SD	4,660	2,782
*P* value (vs BMI < 25)			0.3676
Complication		Hypertension-	Hypertension +
Sample number		144	240
s-PCK1-Ab level	Average	6,105	7,666
	SD	4,927	6,750
*P* value (vs HT-)			0.0198*
Complication		CVD-	CVD +
Sample number		257	18
s-PCK1-Ab level	Average	4,791	4,415
	SD	4,049	2,230
*P* value (vs CVD-)			0.7568
Complication		Dyslipidemia-	Dyslipidemia +
Sample number		237	26
s-PCK1-Ab level	Average	4,955	3,767
	SD	4,154	2,081
*P* value (vs Dyslipidemia-)			0.0941
Life style		Non-smoker	Smoker
Sample number		142	131
s-PCK1-Ab level	Average	4,491	5,015
	SD	2,863	4,834
*P value* (vs Non-smoker)			0.5445
Life style		Alcohol-	Alcohol +
Sample number		160	114
s-PCK1-Ab level	Average	4,560	5,071
	SD	2,842	5,134
*P* value (vs Alcohol-)			0.9975
Medication		Antihypertensive-	Antihypertensive +
Sample number		106	159
s-PCK1-Ab level	Average	4,850	4,660
	SD	4,982	3,169
*P* value (vs Alcohol-)			0.8093

The subjects were divided into two groups as follows: sex (male and female), type-1 and type-2 DM, BMI < 25 and ≥ 25, presence ( +) or absence ( −) of complications of hypertension, CVD, or dyslipidemia, and lifestyle factors (smoking and alcohol intake habits). Antibody levels (Alpha counts) were compared using the Mann–Whitney *U* test. Sample numbers, averages, and SDs of counts and *P* values are shown

*s-PCK1-Ab* Serum phosphoenolpyruvate carboxykinase 1 antibody, *DM* Diabetes mellitus,

*CVD* Cardiovascular disease, *BMI* Body mass index, *SD* Standard deviation

^*^Significant correlations (*P* < 0.05) are indicated in bold

We performed Spearman's correlation analysis to determine the correlation between s-PCK1-Ab levels and the continuous variables of participant parameters such as age, height, weight, BMI, blood test data, and lifestyle factors such as smoking duration (years) and alcohol intake frequency (times/week). The results showed a significant correlation between s-PCK1-Ab levels and age, calcium level, creatine phosphokinase level, platelet number, and blood pressure (Table 5).

**Table 5:** Correlation analysis of serum antibody levels against PCK1 with data on subjects in DM cohort

	*r* value	*P* value
**Age**	**0.1777**	0.0032*
Height (cm)	-0.0947	0.1222
Weight (kg)	-0.1072	0.0766
BMI	-0.0364	0.5515
AST	0.1024	0.0926
ALT	-0.0152	0.8027
LDH	0.0805	0.1913
ALP	0.0691	0.2641
TP	0.0342	0.5905
UA	0.0568	0.3548
UN	0.0602	0.3239
CRE	0.0601	0.3227
T-CHO	0.0434	0.5189
NA	-0.0517	0.4027
K	0.0323	0.6022
CL	0.0411	0.5065
**Ca**	**-0.2075**	0.0312*
G-GTP	0.0353	0.5648
CHE	-0.1264	0.1102
TG	0.0842	0.1669
Creatinin	0.0203	0.7390
eGFR	0.1043	0.0865
**CPK**	**-0.1567**	0.0176*
GLU	0.0480	0.4358
HbA1c	-0.0460	0.4515
LDL-CHO	0.1172	0.0596
GA	-0.1362	0.1098
HDL-CHO	-0.0634	0.3028
WBC	-0.0056	0.9307
RBC	-0.0661	0.3058
**PLT**	**-0.1851**	0.0039*
**Blood pressure**	**0.1571**	0.0092*
Antihypertensive	0.0149	0.8092
Smoking period (year)	0.1263	0.2177
Alcohol Freq (time/w)	0.1445	0.1648

Sample numbers, correlation coefficients (*r* values), and *P* values obtained by Spearman's correlation analysis are shown. Subjects' data used were age, height, weight, body mass index (BMI), aspartate aminotransferase (AST), alanine amino transferase (ALT), lactate dehydrogenase (LDH), alkaline phosphatase (ALP), total protein (TP), uric acid (UA), blood urea nitrogen (BUN), total cholesterol (T-CHO), sodium (Na), potassium (K), chlorine (Cl), calcium (Ca), γ-glutamyl transpeptidase (γ-GTP), cholinesterase (CHE), triglyceride (TG), estimated glomerular filtration rate (eGFR), creatine phosphokinase (CPK), blood sugar (BS), glycated hemoglobin (HbA1c), low-density lipoprotein cholesterol (LDL-C), glycoalbumin (GA), high-density lipoprotein cholesterol (HDL-C), white blood cell number (WBC), red blood cell number (RBC), platelet number (PLT), blood pressure, antihypertensive, smoking duration (year), and alcohol intake frequency (times/w)

^*^Significant correlations (*P* < 0.05) are indicated in bold. DM, diabetes mellitus

## Discussion

During our analysis, the initial SEREX screening identified PCK1 as an antigen as recognized by serum IgG in patients with atherosclerosis, and subsequently, recombinant GST-tagged PCK1 protein of 301 amino acids was purified. Using recombinant PCK1 protein as an antigen, we examined serum antibody levels using AlphaLISA. The results showed that significantly higher s-PCK1-Ab levels were observed exclusively in patients with DM, but not in those with AIS, TIA, and OSAS, compared with those in HDs (Figs. 1a, 2a, b, Tables 1, 2, 3 and 4). Patients with CVD showed a minimal significant difference from HDs. The AUC value of s-PCK1-Abs versus DM was 0.7024 (Fig. 1b). This DM-specific association of s-PCK1-Ab is distinct from previous results that showed that most of the SEREX autoantibodies screened using sera from patients with atherosclerosis were associated with multiple atherosclerosis-related diseases such as AIS and CVD [17, 18]. Our analysis shows that s-PCK1-Abs indicate the presence of DM with a sensitivity and specificity of 36.73% and 95.06%, respectively, by ROC curve analysis. A sensitivity of 36.73% was considered high because the specificity was 95.06%. When the specificity was set to 60%, the sensitivity increased to approximately 65% and 95.06%, respectively.

The comparison of patients' data with the Mann–Whitney U test and Spearman's correlation analysis showed a close correlation between s-PCK1-Ab levels and hypertension (Tables 4 and 5). Antibody levels did not significantly correlate with blood sugar (BS) (*P* = 0.4358), HbA1c (*P* = 0.4515), or glycoalbumin (*P* = 0.1098), which are typical DM markers (Table 5).

PCK1 is an enzyme that converts oxaloacetic acid to phosphoenolpyruvate; there are two isozymes in humans, mitochondrial (PCK 2) and cytosolic (PCK1) PCK [19–21]. PCK1 is involved in gluconeogenesis and is a crucial enzyme in glucose metabolism in the body [22]. Indeed, *PCK1*-knockout mice die early after birth with profound hypoglycemia [23], which was partially rescued by the overexpression of PCK1 in the liver [24]. Therefore, gluconeogenesis under the control of PCK1 in the liver is crucial to avoid hypoglycemia. In contrast, the overexpression of PCK1 in mice leads to diabetes [25]. It has also been reported that a − 232C/G containing promoter of PCK1 showed 5- to 100-fold increased basal expression of PCK1 compared with –232C, and − 232C/G polymorphism of PCK1 has been associated with an increased risk of type 2 DM [26–28]. Glucagon, glucocorticoid, and retinoic acid increased the expression of PCK1, whereas insulin inhibited its expression [29]. Therefore, PCK1 expression increases in the presence of insulin resistance.

Our results showed that s-PCK1-Ab levels were higher in patients with DM than in HDs. Since PCK1 expression seems to increase under diabetic conditions, it might make sense that s-PCK1-Ab levels were higher among patients with DM. One of the major reasons for the development of autoantibodies could be the destruction of lesion tissue, followed by the leakage of intracellular antigenic proteins. Although the leaked proteins may rapidly degrade, repeated leaking and exposure to antigens can tremendously elevate antibody levels. Autoantibody markers are more sensitive than antigen markers. This means that antibody markers can detect very early stages of disease progression [11]. Thus, it is not surprising that antibody markers predict the fate of the disease several years later. Since patients with DM, especially those with insulin resistance, often complicate nonalcoholic fatty liver disease and nonalcoholic steatohepatitis, there is a high chance of PCK1 leaking from live cells, which might produce antibodies against PCK1 [30]. Insulin resistance develops not only in atherosclerotic vascular disease but also in hypertension; therefore, s-PCK1-Ab levels are related to CVD and hypertension.

Apart from the complications and comorbidities of DM, it is intriguing that s-PCK1-Ab levels are related to prognosis. Since the mortality rate of DM is much higher than that of HDs, prognostic markers can be used to detect high-risk patients with poor prognosis among patients with DM. Thus, s-PCK1-Ab may be a useful marker. However, we still do not know why high s-PCK1-Ab levels are related to poor prognosis. One reason for this may be the high incidence of CVD. Indeed, it has been reported that a functional promoter polymorphism in PCK1 is associated with carotid wall thickness [31], which is associated with CVD. PCK1 and PCK2 have also been reported to be critical for the growth of certain cancers [32]. Hence, high levels of s-PCK1-Ab may be related to a high incidence of cancer. The reason for death has not been investigated in all patients with DM who were followed up in this study. Therefore, in future studies, we will identify why high s-PCK1-Ab levels are related to poor prognosis.

This study had some limitations. First, s-PCK1-Abs were measured among the specimens collected at a Japanese university and hospital, which may have biased the specimen population. Second, we do not know the mechanism by which s-PCK1-Abs were increased in patients with DM and hypertension and were related to poor prognosis. Third, the number of patients is small. Therefore, a study with a larger sample and longer duration should be performed in the future to confirm our current findings.

## Conclusions

s-PCK1-Abs are highly sensitive and specific for DM and could be a novel prognostic marker for patients with DM.

### Supplementary Information

**Additional file 1: Supplementary Figure S1.** Sodium dodecyl-sulfate (SDS)–polyacrylamide gel electrophoresis of purified proteins. Purified GST (control) and GST-PCK1 proteins (1 μg) were electrophoresed using SDS–polyacrylamide (10%) gel, followed by staining with Coomassie Brilliant Blue (NacalaiTesque, Kyoto, Japan). The molecular weights of the size markers (Protein Ladder One Plus, NacalaiTesque) are presented on the left. Arrows indicate protein positions: GST: 26 kDa, GST-PCK1: 94 kDa.

## Data Availability

The datasets used and/or analyzed during the current study are available from the corresponding author on reasonable request.

## Abbreviations

CI: 95% Confidence interval
AIS: Acute ischemic stroke
AlphaLISA: Amplified luminescence proximity homogeneous assay-linked immunosorbent assay
AUC: Area under the ROC curve
BMI: Body mass index
CVD: Cardiovascular disease
DM: Diabetes mellitus
GST: Glutathione S-transferase
HD: Healthy donor
OSAS: Obstructive sleep apnea syndrome
PCK: Phosphoenolpyruvate carboxykinase 1
ROC: Receiver operating characteristic
s-PCK1-Ab: Serum anti-PCK1 antibody
SD: Standard deviation
SEREX: Serological identification of antigens by recombinant cDNA expression cloning
TIA: Transient ischemic attack
