# Supplementary material for: Serum anti-PCK1 antibody levels are a prognostic factor for patients with diabetes mellitus
Source: BMC Endocr Disord. 2023 Oct 30;23:239. doi: 10.1186/s12902-023-01491-3 (PMC10614393; doi:10.1186/s12902-023-01491-3)
Supplement: Supplementary file 1 — Additional file 1: Supplementary Figure S1. Sodium dodecyl-sulfate (SDS)–polyacrylamide gel electrophoresis of purified proteins. Purified GST (control) and GST-PCK1 proteins (1 μg) were electrophoresed using SDS–polyacrylamide (10%) gel, followed by staining with Coomassie Brilliant Blue (NacalaiTesque, Kyoto, Japan). The molecular weights of the size markers (Protein Ladder One Plus, NacalaiTesque) are presented on the left. Arrows indicate protein positions: GST: 26 kDa, GST-PCK1: 94 kDa. [file 12902_2023_1491_MOESM1_ESM.pdf]

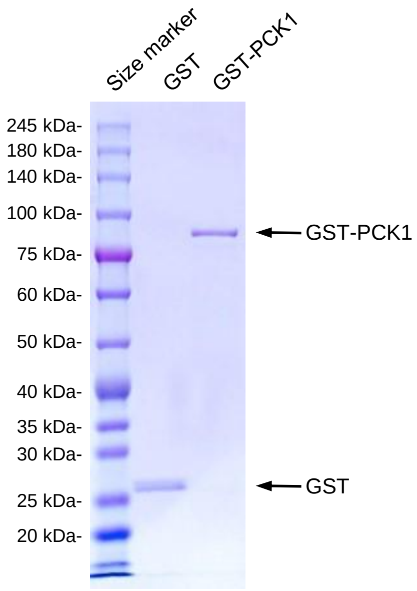

Supplementary Figure S1.
Sodium dodecyl-sulfate (SDS)–polyacrylamide gel electrophoresis of purified proteins. Purified GST (control) and GST-PCK1 proteins (1 μg) were electrophoresed using SDS–polyacrylamide (10%) gel, followed by staining with Coomassie Brilliant Blue (Nacalai Tesque, Kyoto, Japan). The molecular weights of the size markers (Protein Ladder One Plus, Nacalai Tesque) are presented on the left. Arrows indicate protein positions: GST: 26 kDa, GST-PCK1: 94 kDa.
